# Tectonic Deep Anterior Lamellar Keratoplasty in Severe Ocular Chuna Particle Injury in a Child

**DOI:** 10.7759/cureus.41712

**Published:** 2023-07-11

**Authors:** Suman Lata, Aafreen Bari, Tushar Agarwal

**Affiliations:** 1 Ophthalmology, All India Institute of Medical Sciences, New Delhi, IND

**Keywords:** non-healing epithelial defect, tectonic lamellar keratoplasty, ocular chemical injury, ocular trauma, chuna injury

## Abstract

A 10-year-old child presented to eye casualty with pain, watering and photophobia of the left eye for one month. Parents gave a history of chuna (lime) falling inside the eye one month back, following which the patient consulted at a primary health centre. However, there was no relief of symptoms and the patient was referred to a higher centre. On examination under anaesthesia, the eye was full of chuna particles. Removal of all visible chuna particles and the corneal chuna plaque was planned. Intra-operatively, the stromal melt was noted around the corneal chuna plaque extending up to Descemet’s membrane. Microscope-integrated optical coherence tomography (Mi-OCT) guided removal of corneal chuna particles was done. A tectonic deep anterior lamellar keratoplasty (DALK) along with amniotic membrane graft (AMG) and symblepharon ring placement was done. Two weeks post-operatively, the patient was having a persistent epithelial defect. A repeat AMG with a symblepharon ring was done. On one month follow-up, the epithelial defect had healed. This case emphasises the fact that ocular chemical injuries are an emergency requiring urgent and apt management. In cases of severe ocular chuna injury with delayed presentation, removal of all particles, maintenance of globe integrity and ocular surface restoration is a challenging goal. Healing is slow and visual prognosis is generally guarded in such cases.

## Introduction

Chuna particle injury is a very serious and prevalent eye emergency in the developing world due to the tobacco chewing habit of adults and the use of this chemical in households frequently. Chuna or lime is easily available over the counter in the form of small packets and plastic bottles. Unfortunately, the young ones are at risk of contact and exposure to chuna too, owing to common storage at home and vicinity, leading to mild to very severe ocular injuries. This case highlights the severity of chuna-related eye injury and the consequences of late presentation. It is an example of severe negligence by parents and primary eye care provider which leads to corneal stromal melt. This case highlights the grievous nature of the injury, related complications, treatment challenges and outcomes.

## Case presentation

A 10-year-old child presented to ocular emergency with pain, redness, watering, photophobia and diminution of vision in the left eye for a month. The parents gave a history of accidental rupture of chuna packet at home, which fell in his eyes, while he was playing at home. They got primary treatment done at a local centre, where eye wash was given and topical eye medications were prescribed. No surgical intervention was done then. On examination, the visual acuity in the right eye (OD) was 20/20, the left eye (OS) was perception of light (PL), projection of rays (PR) was accurate in all quadrants. On torch light examination, OS appeared to be smaller with a small palpebral aperture, upper and lower lid symblepharon and whitish cornea. 

**Figure 1:**
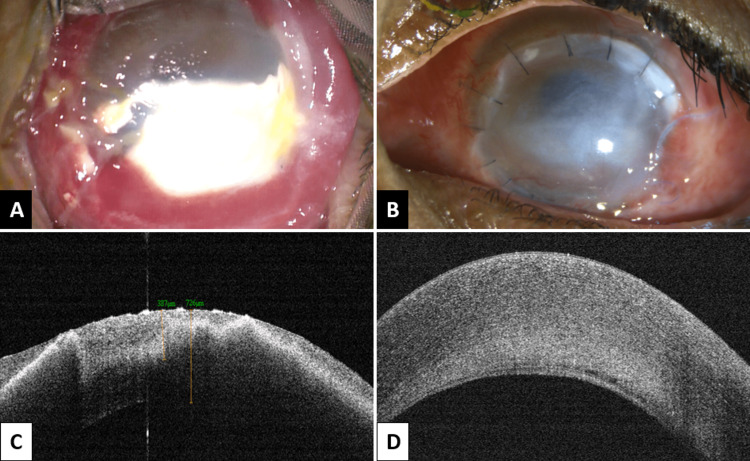
Pre-operative and post-operative clinical image and anterior segment optical coherence tomography (A) Pre-operative image with large chuna particle embedded in the cornea; (B) Post-operative deep anterior lamellar keratoplasty (DALK) with central haze and interrupted 10-0 monofilament-nylon (MNF) sutures; (C) Pre-operative anterior segment optical coherence tomography (AS-OCT) with hyper-reflective chuna particle in the anterior corneal layers and underlying shadowing; (D) Post-operative AS-OCT with well-apposed donor and host corneal layers

The patient was planned for examination under anaesthesia (EUA). Under general anaesthesia, upper lid and lower lid symblepharon were released. The upper and lower fornices were examined. They were full of chuna particles with conjunctival necrosis. A large chuna particle was embedded in the lower half of the cornea with corneal stromal melt. Intra-operatively, Microscope-integrated optical coherence tomography (Mi-OCT) was used to determine the extent and depth of chuna particle embedding into the corneal stroma. It involved more than 80 per cent corneal depth causing a shadowing effect on OCT (Figure 1 A & C).

**Figure 2:**
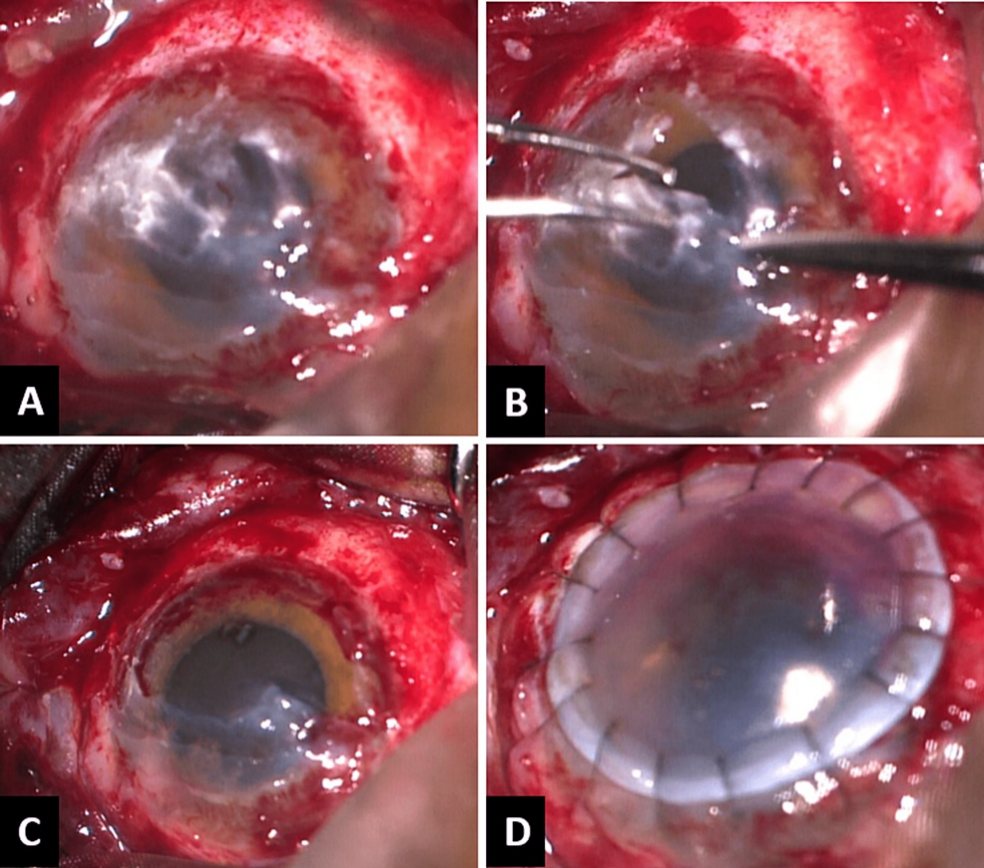
Intra-operative Images of chuna particle removal (A) After gross dissection of chuna particle, hazy posterior stroma with residual chuna particles; (B) Removal of posterior stromal layer exposing the Descemet’s membrane; (C) Bare Descemet’s membrane after removal of all chuna particles; (D) A well-apposed donor tissue to host (Descemet’s membrane) with 16 interrupted monofilament-nylon sutures

After layer-by-layer removal of chuna plaque, stromal tissue embedded with smaller chuna particles was noticed. A decision to continue layer-by-layer dissection and deep anterior lamellar keratoplasty was made after exposing bare Descemet’s membrane layer at the end of dissection. After the removal of donor corneal tissue's Descemet's membrane, it was transferred to the host bed. The graft was secured with sixteen interrupted 10-0 monofilament-nylon sutures (Figure 2 A-D).

The amniotic membrane graft (AMG) was placed over the cornea and into the fornices and secured to the upper and lower lid along with the insertion of a symblepharon ring. A temporary tarsorrhaphy was done. The patient was started on topical antibiotics, steroids, cycloplegics, lubricants, and antiglaucoma medications in post-operative period. Vitamin-C chewing tablets were also prescribed to hasten the healing process. On one week follow-up, the patient was symptomatically better with visual acuity of 1/60 in OS. The donor and host corneal layers were well apposed with no interface fluid or haze. The epithelial defect in donor cornea was not healed at two weeks follow-up. A repeat AMG procedure was done. On EUA at 4 weeks post-operatively, the epithelial defect had healed completely, the fornices were well formed and intraocular pressure (IOP) was well controlled (Figure 1 B & D).

## Discussion

Ocular chemical injuries vary in severity depending on the insulting agent and time of presentation. It is an ophthalmic emergency which requires immediate management. Ocular chemical injuries can have a wide spectrum of presentations from mild-grade ocular burns to total corneal melt, which may have devastating blinding outcomes. The management starts with checking the ocular pH, immediate copious eye wash and grading the chemical burns using Roper Hall or Dua’s classification [1,2]. Chemical burns are one of the most common workplace injuries [3]. The literature review suggested that most chemical injuries are accidental in children, highlighting the need of educating parents about keeping such chemicals out of the reach of kids and thus preventing such accidents [4]. The delayed presentation further makes things worse for the eye and the patient. In a study by Vajpayee et al, the average time of presentation in India was 68.3 days [5]. This delay worsens the prognosis as the patient does not receive any treatment in acute and early phase of injury.

Alkali insult is a more common cause of ocular burns as compared to acids, with chuna being the most common agent [6-8]. Alkali-related ocular injuries have more severe burns and poorer prognosis. It is due to deeper and faster penetration in the cornea as it produces hydroxyl ion on hydrolysis and saponifies the fatty acids in the cell membrane leading to cell lysis which results in more damage.

This case highlights a neglected case of severe chemical injury (lime) involving both superior and inferior fornix with impacted chuna particles in the conjunctiva and chuna plaque embedded in the cornea. Mi-OCT was used to see the extent of stromal involvement by chuna plaque. The chuna plaque was seen to involve deep corneal stroma and caused a shadowing effect. It was further used to delineate the depth of involvement of corneal stroma. As the chuna particles were deep in the corneal stroma, a deeper dissection was required until the Descemet’s membrane. Mi-OCT guided as an important tool to assess the depth of dissection, preserve the integrity of bare Descemet’s membrane and confirm the apposition of donor and host interface [9]. An AMG was used for the restoration of the conjunctival ocular surface, epithelial healing of the donor corneal tissue and preventing symblepharon formation. It is known to reduce limbal stromal inflammation and prevent limbal stem cell deficiency [10]. Thus, there is a need for early presentation of such ocular chemical injuries to an ophthalmologist, especially in such severe cases. Negligence may not only worsen the visual prognosis but the chances of restoration of globe integrity. Mi-OCT is a useful tool to monitor the depth of corneal involvement and guides for better and apt management.

## Conclusions

Early presentation to an ophthalmologist is the most crucial part in managing cases of ocular chemical injury. A thorough eye wash with removal of residual chemical using double eversion technique is of utmost importance. It normalizes the ocular pH and removes the chemical particles decreasing the chances of persistent ocular injury. In severe cases with deeper ocular penetration of chemicals, lamellar or full thickness penetrating keratoplasty may be required for tectonic restoration of ocular integrity. These cases also need long term treatment and monitoring, hence the patient or parents counselling regarding the nature of insult and treatment is crucial.
